# Treatment strategies to prevent and reduce gynecomastia and/or breast pain caused by antiandrogen therapy for prostate cancer: Statement from the DEGRO working group prostate cancer

**DOI:** 10.1007/s00066-020-01598-9

**Published:** 2020-03-12

**Authors:** Pirus Ghadjar, Daniel M. Aebersold, Clemens Albrecht, Dirk Böhmer, Michael Flentje, Ute Ganswindt, Stefan Höcht, Tobias Hölscher, Arndt-Christian Müller, Peter Niehoff, Michael Pinkawa, Felix Sedlmayer, Daniel Zips, Thomas Wiegel, Prostate Cancer Expert Panel of the German Society of Radiation Oncology (DEGRO) and The Working Party Radiation Oncology of the German Cancer Society (DKG-ARO)

**Affiliations:** 1 Department of Radiation Oncology, Charité – Universitätsmedizin Berlin, corporate member of Freie Universität Berlin, Humboldt-Universität zu Berlin, and Berlin Institute of Health, Berlin, Germany, Augustenburger Platz 1, 13353 Berlin, Germany; 2 Inselspital, Bern University Hospital, University of Bern, Bern, Switzerland; 3 Klinikum Nürnberg Nord, Nürnberg, Germany; 4 Universitätsklinikum Würzburg, Würzburg, Germany; 5 Innsbruck Medical University, Innsbruck, Austria; 6 Xcare Praxis für Strahlentherapie Saarlouis, Xcare Gruppe, Saarlouis, Germany; 7 Universitätsklinikum Carl Gustav Carus, Technische Universität Dresden, Dresden, Germany; 8 Universitätsklinikum Tübingen, Tübingen, Germany; 9 Sana Klinikum Offenbach, Offenbach, Germany; 10 MediClin Robert Janker Klinik, Bonn, Germany; 11 Landeskrankenhaus, Universitätsklinikum der Paracelsus Medizinischen Privatuniversität, Salzburg, Austria; 12 Universitätsklinikum Ulm, Ulm, Germany

**Keywords:** Gynecomastia, Breast pain, Prostate cancer, Antiandrogen therapy, Treatment

## Abstract

**Aim:**

To provide an overview on the available treatments to prevent and reduce gynecomastia and/or breast pain caused by antiandrogen therapy for prostate cancer.

**Methods:**

The German Society of Radiation Oncology (DEGRO) expert panel summarized available evidence published and assessed the validity of the information on efficacy and treatment-related toxicity.

**Results:**

Eight randomized controlled trials and one meta-analysis were identified. Two randomized trials demonstrated that prophylactic radiation therapy (RT) using 1 × 10 Gy or 2 × 6 Gy significantly reduced the rate of gynecomastia but not breast pain, as compared to observation. A randomized dose-finding trial identified the daily dose of 20 mg tamoxifen (TMX) as the most effective prophylactic dose and another randomized trial described that daily TMX use was superior to weekly use. Another randomized trial showed that prophylactic daily TMX is more effective than TMX given at the onset of gynecomastia. Two other randomized trials described that TMX was clearly superior to anastrozole in reducing the risk for gynecomastia and/or breast pain. One comparative randomized trial between prophylactic RT using 1 × 12 Gy and TMX concluded that prophylactic TMX is more effective compared to prophylactic RT and furthermore that TMX appears to be more effective to treat gynecomastia and/or breast pain when symptoms are already present. A meta-analysis confirmed that both prophylactic RT and TMX can reduce the risk of gynecomastia and/or breast pain with TMX being more effective; however, the rate of side effects after TMX including dizziness and hot flushes might be higher than after RT and must be taken into account. Less is known regarding the comparative effectiveness of different radiation fractionation schedules and more modern RT techniques.

**Conclusions:**

Prophylactic RT as well as daily TMX can significantly reduce the incidence of gynecomastia and/or breast pain. TMX appears to be an effective alternative to RT also as a therapeutic treatment in the presence of gynecomastia but its side effects and off-label use must be considered.

## Introduction

Androgen deprivation therapy (ADT) is commonly used in metastatic prostate cancer (PCA) or combined with primary radiation therapy (RT) for patients with localized PCA with intermediate- to high-risk features, locally advanced PCA or biochemically recurrent prostate cancer [1–3].

A common side effect of ADT, due to the disturbed balance between estrogens and androgens throughout the body, can be swelling of the male breast called gynecomastia and/or breast pain (mastodynia). Generally, stimulation of estrogen receptors in the breast tissue stimulate growth, while stimulation of androgen receptors inhibits growth. Nonsteroidal antiandrogens like bicalutamide or flutamide block androgen receptors, which through a feedback loop increase the secretion of luteinizing hormone (LH). Increased LH stimulates testosterone secretion, which, however, is then converted to estrogen by peripheral aromatization [4]. As androgen receptors are blocked by nonsteroidal antiandrogens, the increased level of estrogen stimulating the estrogen receptor in breast tissue stimulates growth, leading to gynecomastia and/or breast pain [4].

Gynecomastia and/or breast pain can be observed in up to 85% of patients after therapy with high-dose nonsteroidal antiandrogens, negatively impacting patients’ quality of live (QoL) and treatment compliance [1].

The use of enzalutamide, an androgen receptor signaling inhibitor, besides binding to the androgen receptor inhibiting also DNA binding and coactivator recruitment, has likewise been shown to be associated with a 49% rate of gynecomastia and 21% rate of nipple pain within 2 years [5]. A lower rate of gynecomastia and/or breast pain of only around 13–22% is observed, when combined androgen blockade is used [1].

For apalutamide, a new selective androgen signal inhibitor, gynecomastia is not described as a drug-related side effect.

ADT-related gynecomastia and/or breast pain can be treated by antiproliferative low-dose RT to the breasts. Alternatively, gynecomastia and/or breast pain can be treated by drug intervention using either tamoxifen (TMX) which blocks the estrogen receptor, or theoretically by anastrozole which inhibits the peripheral aromatization of androgens into estrogens. Surgery might also be a treatment option but due to its invasiveness is commonly preserved for those patients were aforementioned treatments have failed [1, 4]. The literature supporting the use of these treatment approaches is reviewed in the following.

## Materials and methods

Complete reports of randomized controlled trials or meta-analysis of RCTs of RT and/or drug interventions to prevent gynecomastia and/or breast pain (prophylactic treatment) or to treat existing gynecomastia and/or breast pain (therapeutic treatment) in patients receiving ADT for prostate cancer were searched in May 2019 using MEDLINE, Current Contents, PubMed, and references from relevant articles. The search strategy included the terms “prostate cancer”, “androgen deprivation therapy”, “hormonal therapy”, “antiandrogen therapy”, “gynecomastia”, “breast pain”, “mastodynia”, “treatment”, alone or in combination.

The primary objective was efficacy to treat gynecomastia and/or breast pain as well as treatment-related toxicities.

Original articles written in English language and published in peer-reviewed journals after the year 2000 were included. Two authors (P.G. and T.W.) selected studies for inclusion.

## Results

### Characteristics of included studies

A total of 8 randomized controlled trials (one trial with two randomizations) and one meta-analysis was identified testing the effect of radiation therapy for ADT-related gynecomastia and/or breast pain. Two randomized controlled trials compared prophylactic RT with no RT or sham RT [6, 7]. Two randomized controlled trials compared prophylactic TMX with different dosage and application schedule [8, 9]. Two trials compared TMX vs. anastrozole or placebo [10, 11]. One trial compared prophylactic TMX vs. therapeutic TMX [12]. One trial compared no prophylaxis, prophylactic RT with prophylactic TMX [13, 14]. Patients from the control arm of the latter trial, who subsequently developed gynecomastia, were randomized to therapeutic RT vs. therapeutic TMX [13, 14].

A meta-analysis included five randomized controlled trials analyzing RT vs. observation or RT vs. TMX, or TMX vs. observation [15].

No randomized trials were found using surgery as treatment modality for gynecomastia and/or breast pain.

### Radiation therapy

The two identified randomized controlled trials are summarized in Table 1 testing the effect of radiation therapy for ADT-related gynecomastia and/or breast pain.

**Table 1:** Characteristics of prospective randomized controlled trials evaluating the role of radiation therapy for ADT-related gynecomastia and/or breast pain

Publication/author	Patients (*N*)	Trial design	Study period	Interventions and results	Side effects and/or QoL
Tyrell et al. [6]	106	Randomized, sham controlled, double blind	11/1999–06/2001	Prophylactic single dose electron beam RT with 10 Gy vs. sham RT. RT reduced gynecomastia 52% vs. 85%; OR, 0.13; 95% CI, 0.04–0.38; *p* < 0.001. Breast pain was similar in both arms (83% vs. 91%; OR, 0.25; 95% CI, 0.05–1.27; *p* *=* 0.221) but severity of pain tended to improve after RT (*p* = 0.043)	RT-related adverse events were experienced by 17 of 52 patients (33%) who received RT (breast/nipple erythema, breast/nipple tenderness, or skin irritation)
Ozen et al. [7]	125	Randomized	06/2003–10/2005	Prophylactic RT using 2 × 6 Gy electron beam vs. no RT RT reduced gynecomastia 15.8% vs. 50.8% while the rate of breast pain was similar being 36.4% vs. 49.2%	Adverse events were mild and observed in 6 or 8 patients per treatment arm

*ADT* androgen deprivation therapy, *QoL* quality of life, *RT* radiation therapy, *OR* odds ratio, *CI* confidence interval, *n.a.* not available

One randomized trial was conducted in a double blind, sham-controlled fashion. One hundred-six prostate cancer patients who underwent bicalutamide treatment were randomized to prophylactic single dose electron beam RT with 10 Gy vs. sham RT [6]. The dose was directed to a 5-cm diameter circle of tissue centered around each nipple and a minimum dose of 90% was required between the skin and the chest wall using 6–12 MeV. RT reduced gynecomastia to rates of 52% vs. 85% (odds ratio [OR], 0.13; 95%, confidence interval [CI], 0.04–0.38; *p* < 0.001) after one year. Breast pain was similar in both arms (83% vs. 91%; OR, 0.25; 95% CI, 0.05–1.27; *p* = 0.221) but developing pain appeared to be less severe after RT (*p* = 0.043) [6].

In another randomized controlled trial in 125 prostate cancer patients who underwent bicalutamide treatment the use of prophylactic electron beam RT using 2 × 6 Gy vs. observation significantly reduced the rate of gynecomastia from 50.8% to 15.8% (*p* < 0.001) after one year, while the incidence of breast pain was not significantly lower (36.4% vs. 49.2%, respectively). RT was directed toward a 5 cm diameter circle of tissue centered on each nipple. The dose was prescribed to the 90% isodose line covering the superficial 3–4 mm of the pectoral muscle using 6 to 12 MeV [7].

### Drug therapy

The five identified randomized controlled trials testing the effect of drug therapy for ADT-related gynecomastia and/or breast pain are summarized in Table 2.

**Table 2:** Characteristics of prospective randomized controlled trials evaluating the role of drug therapy for ADT-related gynecomastia and/or breast pain

Publication/author	Patients (*N*)	Trial design	Study period	Interventions and results	Side effects and/or QoL
Fradet et al. [8]	282	Randomized, placebo controlled	11/2002–06/2003	Patients were randomized to different prophylactic doses of TMX (1, 2.5, 5, 10, or 20 mg/day) or placebo given for 12 months. Increasing doses of TMX decreased incidence of breast events (gynecomastia and/or breast pain) in a dose-dependent manner at the 3‑, 6‑ and 12-month assessments, where breast events were presented in 96.7% or 98.3% after placebo and in 8.8% or 20.6% of patients after 20 mg TMX. At 24 months (i.e., after 12 months of bicalutamide monotherapy), a high incidence of breast events (>90%) was seen in all groups	Dizziness and hot flushes increased by TMX
Bedognetti et al. [9]	80	Randomized, noninferiority	12/2003–2/2006	Patients were randomized to prophylactic daily TMX 20 mg or daily TMX 20 mg for 8 weeks and weekly TMX 20 mg thereafter. After a preplanned interims analysis gynecomastia developed in 31.7% of patients in the daily group and in 74.4% of patients in the weekly group (*p* < 0.0001), and it was more severe in patients who switched to weekly tamoxifen (*p* = 0.001). Mastalgia occurred in 12.2 and 46.1% of patients, respectively (*p* = 0.001)	No difference in treatment-related toxicity
Saltzstein [10]	107	Randomized, placebo controlled	n.a.	Patients were randomized to 3 months of prophylactic daily TMX 20 mg or daily anastrozole 1 mg or placebo. After 3 months bicalutamide was continued for another 9 months. Patients who developed gynecomastia and/or breast pain during this period underwent TMX or anastrozole for 3 months. The incidence of gynecomastia and/or breast pain was 11.8% for TMX, 63.9% for anastrozole and 69.4% for placebo (*p* < 0.0001). Resolution of gynecomastia and breast pain for therapeutically treated patients occurred in 65.4% in the TMX group, 71.8% of patients in the placebo group (who received TMX) and 18.8% of patients in the anastrozole group	Dizziness increased by TMX
Boccardo [11]	114	Randomized, placebo controlled	12/2000–12/2002	Patients were randomized to 48 weeks of prophylactic daily TMX 20 mg or daily anastrozole 1 mg or placebo. Gynecomastia developed in 73% of patients in the bicalutamide group, 10% of patients in the bicalutamide-TMX group, and 51% of patients in the bicalutamide-anastrozole group (*p* = 0.001); breast pain developed in 39%, 6% and 27% of patients, respectively (*p* = 0.006)	Treatment was well tolerated and the incidence of toxicity was higher in the anastrozole arm (69.5% of patients) as compared to placebo (37.5%) or TMX (35.1%)
Serretta [12]	176	Randomized controlled	06/2005–06/2007	Patients were randomized to 1 year daily 20 mg TMX within 1 month from the onset of gynecomastia and/or breast pain (Arm A, therapeutic treatment) or 1 year prophylactic daily 10 mg TMX (Arm B). In Arm A gynecomastia and/or breast pain increased with time with a rate of 39.8%, 57.8%, 69.9% and 78.3% at 3, 6, 9, and 12 months after initiation of bicalutamide. After therapy with TMX gynecomastia and/or breast pain persisted in 27.7% of cases. In arm B, the prevalence of gynecomastia and/or breast pain was 35% after 12 months of therapy. The difference in gynecomastia and/or breast pain between the 2 arms was statistically significant (*p* = 0.0001). The differences in prevalence of gynecomastia and breast pain between the 2 arms both favored TMX prophylaxis (*p* = 0.0001 and *p* = 0.001, respectively)	No significant difference between the trial arms

*ADT* androgen deprivation therapy, *TMX* Tamoxifen, *QoL* quality of life

In a placebo-controlled randomized dose–response study, TMX was tested as prophylactic treatment in 282 prostate cancer patients undergoing bicalutamide therapy [8].

Patients were randomized to different doses of TMX (1, 2.5, 5, 10, or 20 mg/day) or placebo given for 12 months during bicalutamide therapy, followed by 12 months where bicalutamide was given alone. Primary endpoint was breast-related events (gynecomastia and/or breast pain) at 6 months. Increasing doses of TMX were associated with a decreased incidence of breast events in a dose-dependent manner at the 3‑, 6‑ and 12-month assessments. At 6 and 12 months breast events were presented in 96.7% or 98.3% of patients after placebo and in 8.8% or 20.6% of patients after 20 mg TMX. At 24 months (i.e., after 12 months of bicalutamide monotherapy), a high incidence of breast events (>90%) was seen in all groups. There was no evidence of a negative effect of TMX on PSA inhibition at any assessment compared to placebo. An increased incidence of dizziness and hot flushes was observed with prophylactic TMX [8].

In another trial in which prostate cancer patients underwent bicalutamide treatment patients were randomized into either 20 mg daily TMX continuously or 20 mg daily TMX for the first 8 weeks, then at a single weekly dose of 20 mg thereafter for a total of 36 months (or for 24 months in selected patients) in both arms. The trial was stopped early after inclusion of 80 evaluable patients after a planned interims analysis showed the inferiority of the weekly TMX schedule. Breast ultrasonography was used as an objective technique to monitor gynecomastia. There was no difference in treatment-related toxicity between the trial arms. Again, no negative effect on PSA inhibition was observed [9].

Saltzstein et al. included 107 patients with prostate cancer who received bicalutamide therapy in a randomized placebo-controlled trial comparing 3 months of prophylactic daily 20 mg TMX with daily 1 mg anastrozole or placebo with the primary endpoint being gynecomastia and/or breast pain after 3 months. After the 3‑month prophylactic treatment patients continued for another 9 months with the bicalutamide treatment [10]. When gynecomastia and/or breast pain was observed during this period, TMX or anastrozole where given again for 3 months or for patients of the placebo group 20 mg of daily TMX was applied. The incidence of gynecomastia and/or breast pain was 11.8% for TMX, 63.9% for anastrozole and 69.4% for placebo, respectively (*p* < 0.0001). Interpretation of the results at 6, 9, and 12 months after randomization was confounded by the retreatment of some patients with TMX or anastrozole for gynecomastia/breast pain developing before these timepoints, and therefore not presented. The number of patients actively retreated for gynecomastia/breast pain in the TMX, anastrozole, and placebo groups was 26 (76.5%), 32 (88.8%), and 32 (88.8%), respectively. Resolution of gynecomastia and breast pain occurred in 65.4% of patients in the TMX group, 71.8% of patients in the placebo group (who received TMX) and 18.8% of patients in the anastrozole group [10]. The incidence of treatment-related dizziness appeared to be higher in the TMZ arm of the trial. Although serum testosterone levels in the TMX group were higher than those in the placebo group during the prophylactic phase of the clinical trial, any adverse impact of combination therapy on cancer control as assessed by PSA levels could not be observed during the trial [10].

In a placebo-controlled randomized trial on prostate cancer patients who received bicalutamide therapy, 114 patients were randomized into 48 weeks of prophylactic daily 20 mg TMX, or daily 1 mg anastrozole or placebo. Gynecomastia developed in 73% of patients in the bicalutamide group, 10% of patients in the bicalutamide–TMX group, and 51% of patients in the bicalutamide–anastrozole group (*p* = 0.001); breast pain developed in 39%, 6%, and 27% of patients, respectively (*p* = 0.006). Treatment was well tolerated and the incidence of toxicity was higher in the anastrozole arm (69.5% of patients) as compared to placebo (37.5%) or TMX (35.1%). There was no major difference among groups regarding PSA response [11].

Serretta et al. conducted a randomized controlled trial on 176 patients with prostate cancer undergoing bicalutamide therapy. Patients were randomized to either 1 year daily 20 mg TMX within 1 month from the onset of gynecomastia and/or breast pain (Arm A, therapeutic treatment) or 1 year prophylactic daily 10 mg TMX (Arm B). In Arm A gynecomastia and/or breast pain increased with time with a rate of 39.8%, 57.8%, 69.9%, and 78.3% at 3, 6, 9, and 12 months after initiation of bicalutamide. After therapy with TMX gynecomastia and/or breast pain persisted in 27.7% of cases. In arm B, the prevalence of gynecomastia and/or breast pain was 35% after 12 months of therapy. Although TMX was quite effective in the majority of patients of arm B, the action of the drug was slow, with only 18.5% of patients having a clinical response after 3 months of therapy. After 6 months of TMX therapy, the response rate was 43.8%, and many other patients required up to 12 months of administration. The difference in gynecomastia and/or breast pain between the two arms was statistically significant (*p* = 0.0001). The differences in prevalence of gynecomastia and breast pain between the two arms both favored TMX prophylaxis (*p* = 0.0001 and *p* = 0.001, respectively). There was no difference in toxicity observed between the trial arms and there was no difference in PSA control between the trial arms [12].

### Radiation therapy vs. drug therapy

Table 3 summarized the identified randomized controlled trial comparing radiation therapy and drug therapy with two randomizations for ADT-related gynecomastia and/or breast pain.

**Table 3:** Characteristics of prospective randomized controlled trials comparing radiation therapy and tamoxifen for ADT-related gynecomastia and/or breast pain

Publication/author	Patients (*N*)	Trial design	Study period	Interventions and results	Side effects and/or QoL
Di Lorenzo et al. [13]	102	Randomized controlled	01/2002–02/2004	Patients were randomized no additional treatment (control arm) or prophylactic daily 10 mg TMX over 24 weeks or prophylactic low dose RT using 1 × 12 Gy. Patients from the control arm who developed gynecomastia and/or breast pain were subsequently randomized to TMX or RT. Of the control group 67% of patients developed gynecomastia compared with 8% after prophylactic TMX and 34% after prophylactic RT. Breast pain was more frequent in the control group than after TMX or RT (58% vs 7% and 30%, respectively). Differences were significant (*p* = 0.001 or *p* = 0.01). In patients of the control group who had gynecomastia and/or breast pain a significant decrease in symptoms was achieved in those receiving TMX (*p* = 0.05) For further information compare below (Perdonà et al.)	There were no significant differences between the groups with respect to toxicity
Perdonà et al. [14]	151	Randomized controlled	01/2002–02/2004	Same trial as Di Lorenzo et al., published with a larger number of patients but basically the same results	There were no significant differences between the groups with respect to toxicity

*ADT* androgen deprivation therapy, *QoL* quality of life, *RT* radiation therapy

A randomized controlled trial on 102 prostate cancer patients who underwent bicalutamide treatment randomized patients in a control arm or to receive prophylactic daily 10 mg TMX over 24 weeks or prophylactic RT using 1 × 12 Gy. RT was administered as an electron beam directed to irradiate a 5 cm diameter circle of tissue centered around each nipple and was designed to deliver a minimum dose of 90% between the skin and the chest wall. An appropriate electron energy of 6–12 MeV was selected to cover the depth of tissue.

Patients from the control arm who developed gynecomastia and/or breast pain were subsequently randomized to TMX or RT [13].

Of the control group 67% of patients developed gynecomastia compared with 8% after prophylactic TMX and 34% after prophylactic RT. Regarding the power of the study, prophylactic TMX and prophylactic RT both reduced expected frequency by 50% or more and were therefore effective. However, breast pain was more frequent in the control group than after TMX or RT (58% vs 7% and 30%, respectively). Differences were significant (*p* = 0.001 and *p* = 0.01). In patients of the control group who had gynecomastia and/or breast pain a significant decrease in symptoms was achieved in those receiving TMX (*p* = 0.05). There was no difference of treatment-related toxicity or PSA control between the two arms [13].

Data from the latter trial were published again but with more included patients and with similar main results [14]. The 35 patients allocated in the control arm (initial randomization) who developed gynecomastia and/or breast pain were subsequently randomly assigned to TMX (*n* = 17) or RT (*n* = 18). In this subgroup, TMX significantly reduced the frequency of gynecomastia (*p* = 0.02). After 6 months and 9 months, gynecomastia was recorded in 2 of 17 patients allocated TMX compared with 10 of 18 allocated RT. Of these 35 patients, 29 developed concomitant gynecomastia and breast pain, and a higher reduction in pain was recorded in the TMX group than in the RT group (*p* = 0.045). After 6 months and 9 months, breast pain was reported in 4 of 14 patients assigned TMX compared with 12 of 15 assigned RT.

A recent meta-analysis included five randomized trials [6–8, 11, 14] with a total of 777 patients to explore the effects of prophylactic low-dose RT or TMX for patients who underwent ADT for prostate cancer [15]. Pooled results from the trials comparing RT vs. observation showed a significant reduction in the incidence of gynecomastia and breast pain rates in patients treated with RT (OR, 0.21; 95% confidence interval [CI], 0.12–0.37, *p* < 0.0001, and OR, 0.34; 95% CI 0.20–0.57, *p* < 0.0001, respectively). Use of RT resulted in an absolute risk reduction of 29.4 and 19.9%, with a number needed to treat of 3.4 and 5 to avoid one case of gynecomastia and breast pain, respectively. In comparison, pooled results from trials comparing TMX vs. observation likewise showed a statistical benefit for breast pain and gynecomastia in favor of TMX arms (OR, 0.04; 95% CI, 0.02–0.08, *p* < 0.0001 and OR, 0.07; 95% CI, 0.0–0.14, *p* < 0.00001). TMX resulted in a larger absolute risk reduction of 64.1% and 47.6%, with a number needed to treat to avoid one case of gynecomastia and breast pain of 1.56 and 2.1, respectively.

Considering adverse effects, these were reported in three included trials with a total population of 330 patients within the meta-analysis. The incidence of adverse effects (erythema, pruritus or hyperpigmentation) were highest in the RT arms (45/155; 29%) compared with observation arms (48/175; 27.4%), resulting in an absolute risk increase to harm of 1.6% and number needed to harm of 62.5. On the other hand, the incidence of adverse effects in TMX arms was higher (mostly dizziness and hot flushes) compared to the observation arms (47/121; 38.8% vs. 44/151; 29%) with an absolute risk increase to harm of 9.8% and a number needed to harm of 10 [13]. The authors concluded that TMX was two times more effective in preventing gynecomastia; however, low-dose RT represents an effective and safe treatment option, to take into account mainly in patients with cardiovascular risk factors or thrombotic diathesis [15].

## Discussion

The incidence of gynecomastia and/or breast pain after treatment with nonsteroidal antiandrogens or more recently also with enzalutamide is high and effective prophylactic and therapeutic treatments are important. Prophylactic RT (1 × 10–12 Gy or 2 × 6 Gy) is generally well tolerated and can significantly reduce gynecomastia and to a lesser extent breast pain. Secondary cancers are generally a risk after every RT; however, the risk after usage of lower-doses, usually used for treatment of benign diseases, renders the development of second cancers, to the best of our knowledge, very unlikely [16]. Likewise, in a registry-based study on prophylactic RT of the breast in prostate cancer patients, the risk of second cancers was not increased [17].

Daily 20 mg TMX is also a well-established approach and appears to be more effective than RT for prophylaxis and therapeutic treatment for gynecomastia and/or breast pain [13–15]. TMX, however is associated with a well-documented rate of side effects, mostly dizziness [8, 10] and hot flushes [8], whereby the latter might be additive to those produced by ADT, which would adversely affect tolerability of ADT. In addition, the risk of thromboembolic events is significantly increased in older (≥71 years) male patients who undergo TMX treatment for breast cancer [18]. Regarding the risk of secondary cancers due to TMX treatment, in females an increase of endometrial cancers is well-documented, long-term data for prostate cancer patients receiving TMX is not available to estimate the risk of secondary cancers in men. There was no evidence in any of the randomized controlled trials that there is a negative effect of TMX on PSA control, at any assessment compared to placebo [8–12].

The resolution rate for gynecomastia is strictly related to the duration of therapy with bicalutamide [14]. Resolution of gynecomastia and breast pain occurred in round about two thirds of patients who were therapeutically retreated with 3 months of 20 mg daily TMX, or in patients with manifest gynecomastia and/or breast pain who were treated with 3 months of 20 mg daily TMX for the first time [10].

As expected, based on the pharmacologic mechanisms involved, the benefits of prophylactic TMX 20 mg do not persist once therapy was discontinued [10]. Therefore, it is probably required to remain on TMX throughout the course of ADT treatment to maintain a prophylactic effect [8]. Notably, the use of TMX in prostate cancer patients is an off-label use and liability issues may apply for prescribing doctors.

Although low-dose breast RT is usually well tolerated and allows patients to avoid year-long exposition to TMX, late cardiac effects and secondary malignant disease might be of some concern following the delivery of this treatment, especially in the individuals with longer life expectancies, like those who are candidates to receive bicalutamide as an adjuvant treatment [19]. This can however be reduced to a minimum when the appropriate electron energy is chosen.

A comparative histologic examination of breast tissue one year after RT with 12.5 Gy (3 fractions) and normal tissue without previous RT found a significantly improved but not completely inhibited diethylstilboestrol-induced gynecomastia [20]. Gynecomastia can be characterized into two distinct subtypes (type I, increased number of ducts and marked proliferation of ductal epithelia; and type II, minimal proliferation but changes to the stroma and structural components of the breast) [21], it is possible that RT only prevents the proliferative change (type I). Thus, more studies using different schedules of fractionation achieving higher biological doses with conformal RT techniques should be performed to further reduce the incidence of gynecomastia and/or breast pain without increasing the risk of toxicity associated with RT [15, 22].

For clinical practice, besides the comparative effectiveness of TMX and RT also side effects have to be considered. Although RT appears to be less effective than TMX as prophylactic treatment, side effects of RT are only modest and of short duration, whereas the side effects of TMX are usually lasting throughout the whole course of therapy. Patients need to be counseled well regarding this and it might also be an option to start prophylactic RT and switch to TMX, when RT should turn out to be not effective enough.

Due to the associated high incidence of gynecomastia and/or breast pain, prophylactic treatment should only be offered to patients undergoing treatment with nonsteroidal antiandrogens or enzalutamide but not to patients undergoing treatment with other forms of ADT. Currently, gynecomastia is not an issue with the new antiandrogen apalutamide.

Unfortunately, there is no evidence from randomized trials available regarding different fractionation schedules of RT neither as a prophylactic or therapeutic treatment. Today, besides the established 1 × 10–12 Gy single shot treatment, or the 2 × 6 Gy schedule, commonly hypofractionated schedules with 3–5 fractions of 3 Gy are applied, as recently reviewed by the guideline for radiotherapy of benign diseases published by the German Society for Radiation Oncology (DEGRO) and others [23, 24].

For therapeutic treatment of existing gynecomastia and/or breast pain again, TMX is more effective than RT; however, treatment-associated side effects and their duration also have to be considered in decision making and discussed with patients.

For therapeutic RT of existing gynecomastia and/or breast pain even higher doses of around 30–40 Gy total dose are used and recommended [23], but there is no data available supporting this strategy and more data is needed to confirm that this higher dose approach is associated with increased effectivity.

Anastrozole has been shown to be ineffective for the prophylactic treatment of gynecomastia and/or breast pain and should therefore not be used [10, 11].

Long-standing gynecomastia resulting from the fibrotic changes in the adipose tissue appears to be best managed by surgery [25]. However, breast surgery can be associated with complications such as doughnut deformity, nipple necrosis, nipple flatting, and loss of sensation [26].

## Conclusions

Prophylactic RT as well as daily TMX can significantly reduce the incidence of gynecomastia and/or breast pain. TMX appears to be an effective alternative to RT also as a therapeutic treatment in the presence of gynecomastia but its side effects and off-label use must be considered.
